# Small G proteins in peroxisome biogenesis: the potential involvement of ADP-ribosylation factor 6

**DOI:** 10.1186/1471-2121-10-58

**Published:** 2009-08-17

**Authors:** Erin A Anthonio, Chantal Brees, Eveline Baumgart-Vogt, Tsunaki Hongu, Sofie J Huybrechts, Patrick Van Dijck, Guy P Mannaerts, Yasunori Kanaho, Paul P Van Veldhoven, Marc Fransen

**Affiliations:** 1 Department of Molecular Cell Biology, Catholic University of Leuven, Herestraat 49, Leuven, Belgium; 2 Institute for Anatomy and Cell Biology II, Justus-Liebig University, Aulweg 123, Giessen, Germany; 3 Department of Physiological Chemistry, Institute of Basic Medical Sciences, University of Tsukuba, 1-1-1 Ten-nohdai, Tsukuba, Japan; 4 Department of Molecular Microbiology, VIB, Leuven, Belgium; 5 Department of Biology, Catholic University of Leuven, Kasteelpark Arenberg 31, Leuven, Belgium

## Abstract

**Background:**

Peroxisomes execute diverse and vital functions in virtually every eukaryote. New peroxisomes form by budding from pre-existing organelles or *de novo *by vesiculation of the ER. It has been suggested that ADP-ribosylation factors and COPI coatomer complexes are involved in these processes.

**Results:**

Here we show that all viable *Saccharomyces cerevisiae *strains deficient in one of the small GTPases which have an important role in the regulation of vesicular transport contain functional peroxisomes, and that the number of these organelles in oleate-grown cells is significantly upregulated in the *arf1 *and *arf3 *null strains compared to the wild-type strain. In addition, we provide evidence that a portion of endogenous Arf6, the mammalian orthologue of yeast Arf3, is associated with the cytoplasmic face of rat liver peroxisomes. Despite this, ablation of Arf6 did neither influence the regulation of peroxisome abundance nor affect the localization of peroxisomal proteins in cultured fetal hepatocytes. However, co-overexpression of wild-type, GTP hydrolysis-defective or (dominant-negative) GTP binding-defective forms of Arf1 and Arf6 caused mislocalization of newly-synthesized peroxisomal proteins and resulted in an alteration of peroxisome morphology.

**Conclusion:**

These observations suggest that Arf6 is a key player in mammalian peroxisome biogenesis. In addition, they also lend strong support to and extend the concept that specific Arf isoform pairs may act in tandem to regulate exclusive trafficking pathways.

## Background

Peroxisomes are a diverse group of organelles which accommodate many activities related to lipid metabolism. In mammals, these organelles harbor a number of essential metabolic functions including ether phospholipid biosynthesis, fatty acid α – and β-oxidation, and glyoxylate detoxification [1]. The size, number and enzyme content of peroxisomes can be affected by various environmental, metabolic, and developmental factors [2-4]. Peroxisomes are thought to arise *de novo *from the endoplasmic reticulum (ER) [5-8] or multiply by fission of pre-existing peroxisomes [3,9,10]. The relative contribution of both pathways to the total number of peroxisomes in wild-type cells is not yet clear [7,10,11], and the molecular mechanisms governing these processes are only beginning to be unraveled [12].

Studies in different model systems have demonstrated that the Pex11p family of peroxisomal membrane proteins (PMPs), a select set of dynamin-related proteins (DRPs), and Fis1 and Mff – two putative adaptors for DRPs – coordinately control the shape, size and number of peroxisomes in a cell [3,11,13-15]. The Pex11 proteins are supposed to be involved in the elongation and tubulation of pre-existing peroxisomes, whereas the DRPs, Fis1, and Mff are thought to catalyze the final fission event [3,11,15]. It has been put forward also that ADP-ribosylation factors and coatomer proteins are involved in the biosynthetic process of the peroxisomal membrane [16]. Arguments in favor of this hypothesis are the observations that (i) Arf1, Arf6, and COPI coatomer proteins can bind to highly purified rat liver peroxisomes [17-19], (ii) expression of a temperature-sensitive ε-COP subunit in CHO cells at non-permissive temperature exhibited a dramatic change in peroxisome morphology [17], and (iii) the *S. cerevisiae *orthologues of mammalian Arf1 (ScArf1) and Arf6 (ScArf3) regulate peroxisome division up and down, respectively [19]. Also, peroxisomal Arf/COPI might be involved in the retrieval of cargo from peroxisomes back to the ER [12,16,20]. The observation that a dominant-negative mutant of Arf1 inhibits the transport pathway of tomato bushy stunt virus 33 kDa replication protein from peroxisomes to the ER in *Nicotiana tabacum *cells supports this hypothesis [21].

Arf1 and Arf6 are – together with Sar1 – the most comprehensively studied proteins of the ADP-ribosylation factor (ARF) family [22]. Members of this group of small GTPases are believed to control vesicular trafficking and organelle structure by recruiting coat proteins, to function as regulators of phospholipid metabolism, and to modulate cytoskeletal organization [23,24]. Arf1 has been shown to function in the GTP-dependent recruitment of COPI coatomer to budding transport vesicles that move from the Golgi to the ER [25]. In addition, it has been shown that ARF1-GTP regulates the recruitment of clathrin to late Golgi and endosome compartments through binding with heterotetrameric adaptor protein complexes as well as monomeric Golgi-localized γ-ear-containing ARF-binding proteins [26]. Arf6, a protein predominantly associated with the plasma membrane, is thought to regulate endosomal membrane delivery and traffic, membrane raft trafficking, membrane recycling during phagocytosis, and actin remodeling at the cell periphery [23,24]. In this study, we dissect the potential role of small GTPases, and especially of Arf6, in peroxisome biogenesis.

## Results

### Yeast cells deficient in Arf1 or Arf3 exhibit a pronounced increase in peroxisome number on oleate compared to wild-type cells

**Figure 1:**
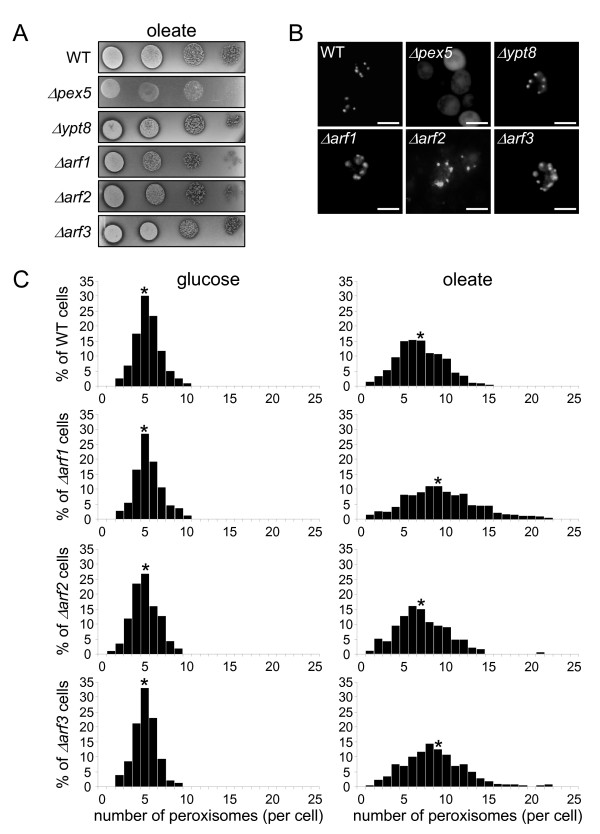
**Phenotypic analysis of various *S. cerevisiae *(deletion) strains**. Serial dilutions of wild-type (WT) yeast cells (strain BY4741) and yeast cells deficient in Pex5p (Δ*pex5*), Ypt8 (Δ*ypt8*), Arf1 (Δ*arf1*), Arf2 (Δ*arf2*), or Arf3 (Δ*arf3*) expressing EGFP-PTS1 were spotted onto plates with oleate as a sole carbon source. (A) The plates were subsequently incubated at 30°C for seven days, and oleate consumption was scored by halo formation. (B) Two days later, the subcellular distribution pattern of EGFP-PTS1 was visualized by fluorescence microscopy. The scale bar represents 5 μm. (C) The number of peroxisomes per cell was counted in randomly selected cells. The mean number of peroxisomes per cell is indicated by an asterisk. At least 100 glucose-grown and 400 oleate-grown cells were scored.

In view of a continuously increasing number of studies reporting that the ER may provide peroxisomes with lipids and some membrane proteins in the form of a preperoxisomal structure [10,27], we investigated whether or not yeast strains deficient in one of the small GTPases that function as a molecular switch in protein trafficking contained functional peroxisomes. As such, all viable null strains of the Rab and the Sar1/Arf families [28] (see Methods for details) were tested for their ability to consume oleate as a sole carbon source, a growth condition requiring functional peroxisomes [29]. A wild-type strain and a strain lacking Pex5p – the import receptor for peroxisomal matrix proteins containing a C-terminal peroxisomal targeting signal – were included as appropriate controls, and oleate consumption was scored by halo formation. None of the yeast strains deficient in one of the small GTPases displayed significant changes in halo formation compared to the wild-type strain (the results of four representative strains are shown in Figure 1A). This observation indicates that the corresponding GTPases are not essential for peroxisome biogenesis in yeast. This conclusion is in line with our observations that none of these strains displayed a detectable peroxisomal sorting defect for the peroxisomal matrix protein marker EGFP-PTS1 (Figure 1B) or the peroxisomal membrane protein marker Pex11p-EGFP (data not shown). Interestingly, the average number of peroxisomes in oleate-grown cells was substantially increased in the Δ*arf1 *and Δ*arf3 *strains compared to the wild-type strain (Figure 1C). To determine whether or not these changes were significant, the values obtained for the wild-type and *Arf*-null strains were analyzed more thoroughly. A one-way ANOVA showed significant differences among the groups (p-value = 0.0001). A subanalysis by the Tukey-Kramer multiple comparisons procedure revealed that the peroxisome number in oleate-grown Δ*arf1 *and Δ*arf3 *yeast cells did indeed significantly differ from that in oleate-grown wild-type cells (both p-values < 0.0001). By contrast, there was no significant pairwise difference between the peroxisome numbers in oleate-grown Δ*arf2 *and wild-type cells (p-value = 0.8733). Summarized, these observations indicate that, at least under certain growth conditions, Arf1 and Arf3 may be involved in the regulation of peroxisome number in *S. cerevisiae*. Interestingly, the construction and analysis of a double Δ*arf1Δarf3 *deletion mutant revealed a cumulative effect of each individual deletion when the cells were grown on oleate (see Additional file 1).

### Copurification of Arf proteins with rat liver peroxisomes

**Figure 2:**
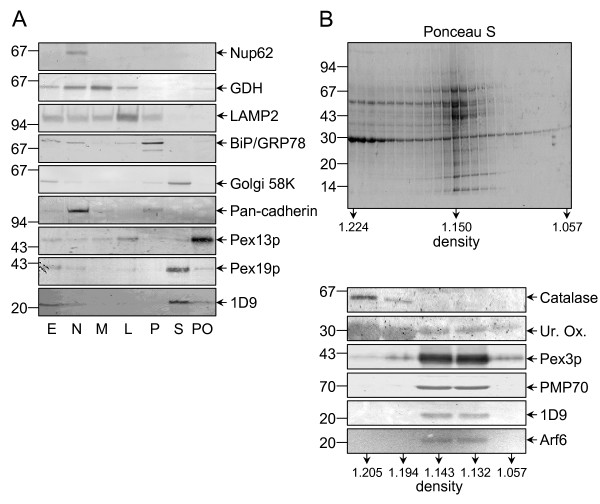
**Arf6 copurifies with the peroxisomal membrane fractions**. (A) Rat liver homogenates were fractionated into a postnuclear (E), a nuclear (N), a heavy mitochondrial (M), a light mitochondrial (L), a microsomal (P), and a cytosolic (S) fraction. A peroxisome-enriched fraction (PO) was obtained by centrifugation of the L-fraction on a Nycodenz step gradient (see Methods). Equal amounts of protein were subjected to SDS-PAGE, transferred to nitrocellulose, and immunoblotted with antibodies raised against the nuclear pore complex protein p62 (Nup62), mitochondrial glutamate dehydrogenase (GDH), lysosomal-associated membrane protein 2 (LAMP2), the ER-resident chaperone immunoglobulin binding protein (BiP/GRP78), the microtubule-binding peripheral Golgi membrane 58 kDa protein (Golgi 58 K), the plasma membrane protein pan-cadherin (Pan-cadherin), the peroxisomal membrane protein Pex13p (Pex13p), peroxisomal biogenesis factor 19 (Pex19p), or a linear epitope found in Arf proteins (1D9). Note that the majority of Golgi 58 K is soluble after fractionation, and that Pex19p is a predominantly cytosolic, partly peroxisomal protein. (B) Six milligrams of total protein from the peroxisomal fraction was processed for floatation centrifugation in an alkaline sucrose gradient (see Methods). The fractions were collected from the bottom, processed for SDS-PAGE, transferred to a nitrocellulose membrane, and stained for total protein with Ponceau S (upper panel). A select set of fractions was immunoblotted with anti-Arf 1D9 or antibodies against Arf6 or peroxisomal matrix (catalase), core (urate oxidase), or membrane proteins (Pex3p, PMP70) (lower panels). The density (in g/ml) of the gradient fractions and the migration of the molecular mass markers (their masses expressed in kDa) are indicated.

**Figure 3:**
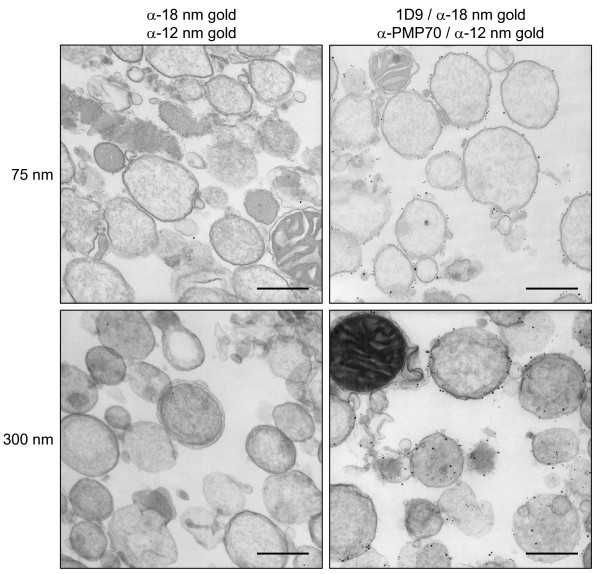
**Electron microscopic localization of 1D9-immunoreactive proteins in prestained epoxy-embedded preparations of rat liver peroxisomes**. Nycodenz-purified rat liver peroxisomes were processed for pre-embedding double immunoelectron microscopy (see Methods). Rabbit anti-PMP70 (α-PMP70) and mouse monoclonal 1D9 antibodies were used as primary antibodies, and 12 nm gold-conjugated anti-rabbit IgG (α-12 nm gold) and 18 nm gold-conjugated anti-mouse IgG (α-18 nm gold) as labels. The primary antibodies were omitted from samples used as negative control (left column). The images were obtained from 75 nm (upper panels) and 300 nm thick (lower panels) sections. The original magnification was 27,800-fold; the scale bar represents 500 nm.

**Figure 4:**
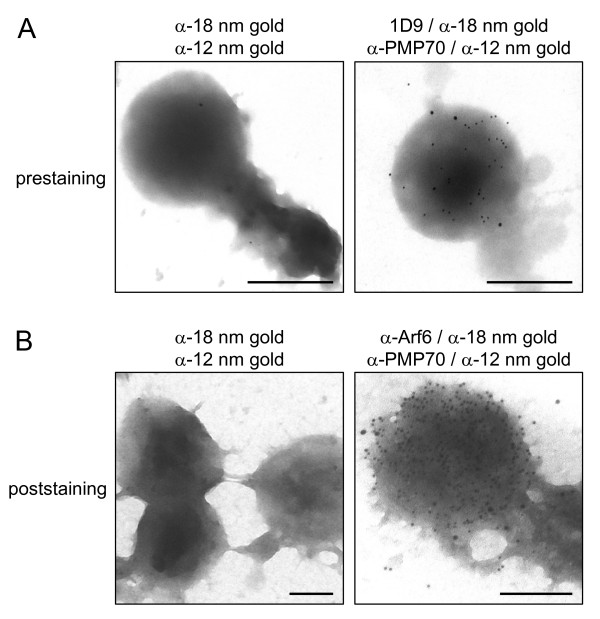
**Electron microscopic localization of 1D9- and anti-Arf6-immunoreactive proteins in Nycodenz-purified peroxisomal fractions immobilized on poly-L-lysine-coated grids**. (A) Prestained organellar fraction (see Methods). Rabbit anti-PMP70 (α-PMP70) and mouse monoclonal 1D9 were used as primary antibodies, and 12 nm gold-conjugated anti-rabbit IgG (α-12 nm gold) and 18 nm gold-conjugated anti-mouse IgG (α-18 nm gold) as labels. (B) Poststained organellar fraction (see Methods). Rabbit anti-PMP70 (α-PMP70) and mouse anti-Arf6 (α-Arf6) were used as primary antibodies, and the same secondary antibodies were used as in panel A. Negative controls were included in parallel in which the primary antibodies were omitted (left panels). The original magnification was 60,000-fold (upper panels), 27,800-fold (lower left panel), or 46,460 fold (lower right panel); the scale bar represents 500 nm.

**Figure 5:**
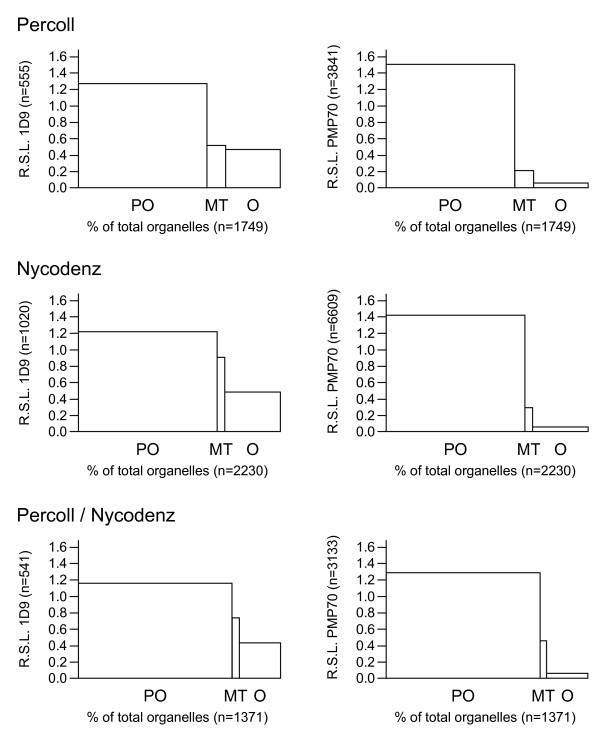
**Organellar distribution of 1D9- and anti-PMP70-immunoreactive proteins in enriched peroxisomal rat liver fractions**. Percoll-, Nycodenz- and Percoll/Nycodenz-purified peroxisomal fractions were processed for pre-embedding double immunoelectron microscopy using anti-Arf 1D9 (18 nm gold grains) and anti-PMP70 (12 nm gold grains) antibodies (see legend to Figure 3). Epon-embedded fractions were quantitatively analyzed for the density of labeling on organellar membranes. The results are expressed as relative specific labeling (R.S.L.) versus percentage of total cell organelles. R.S.L. is hereby defined as the percentage of total gold particles present on a particular organelle divided by the corresponding percentage of total organelles. R.S.L. values greater than one indicate enrichment of the labeling in that specific fraction. The combined total number of cell organelles (x-axis) and gold particles (y-axis) is indicated. Note that the purity of the peroxisomal Percoll, Nycodenz, and Percoll/Nycodenz fractions was 60%, 70%, and 77%, respectively. Abbreviations: PO, peroxisomes (vesicular structures labeled with anti-PMP70 antibodies); MT, mitochondria (double membrane-bound organelles with cristae); O, other vesicles (vesicular structures which could not be unambiguously identified as peroxisomes or mitochondria).

In order to investigate whether or not any endogenous Arf proteins are associated with mammalian peroxisomes, we probed rat liver subcellular fractions with 1D9, a mouse monoclonal antibody that binds to a linear epitope found in Arf proteins but not in other GTP-binding proteins [30]. These experiments revealed the presence of a weak but detectable 21 kDa 1D9-immunoreactive band in the purified peroxisomal fraction (Figure 2A). By performing membrane floatation experiments and employing Arf-specific antibodies, we could show that this protein was membrane-associated and most likely Arf6 (Figure 2B). Consistent with this identification is the observation that antibodies towards Arf1, Arf2, Arf3, and/or Arf4, but not Arf6, did not yield a signal above background (data not shown). Note that the anti-Arf6 antibody employed in this study does not cross-react with Arf1, Arf3, or Arf4 (see Additional file 2, panels B and C), and is only yielding a detectable signal for Arf5 when this protein is expressed in the high-nanogram to low-microgram range (see Additional file 2, panels B and C). Arf2 was not included in these experiments, as this protein – which is absent in humans – is 96% identical (at the amino acid level) to Arf1 (see Additional file 2, panel A). The results obtained with the anti-Arf5 antibody were not conclusive. That is, a very weak signal could be observed, but only after very long exposure times (data not shown). However, as the epitope recognized by the monoclonal antibody directed against Arf5 is unknown, we can not exclude the possibility of some cross-reactivity with Arf6. Finally, as (i) subcellular fractionation cannot exclude with absolute certainty that a particular protein is located in a contaminating organelle that co-purifies with the organelle under study, and (ii) the peroxisomal fractions employed also contained small amounts of glutamate dehydrogenase (mitochondria), BiP/GRP78 (endoplasmic reticulum), and pan-cadherin (plasma membrane) (Figure 2A), we subjected peroxisomal fractions with different degrees of purity to immunoelectron microscopy. These experiments established that the anti-Arf 1D9 (Figures 3, 4A, and 5) and anti-Arf6 (Figure 4B) antibodies specifically recognized a protein on the outer aspect of the peroxisomal membrane, and not on the membrane of a contaminating organelle.

### Arf6 is not essential for peroxisome biogenesis in mouse hepatocytes

**Figure 6:**
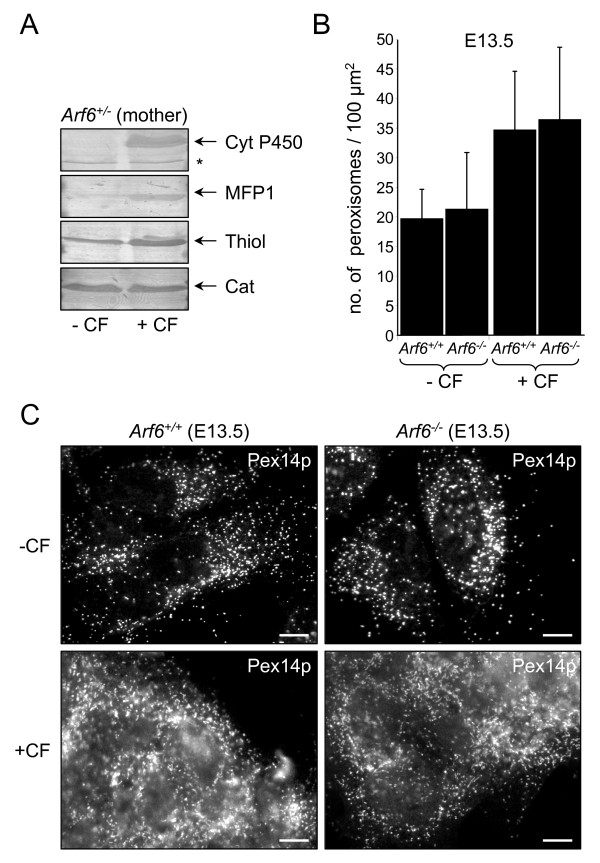
**Arf6 is not essential for peroxisome biogenesis in fetal mouse hepatocytes from control and clofibrate-treated pregnant mice**. (A) Protein (25 μg), present in liver postnuclear supernatants from control (-CF) and clofibrate-treated (+CF) pregnant *Arf6*^+/- ^mice (see Methods), was subjected to SDS-PAGE, transferred to PVDF, and immunoblotted with antibodies against cytochrome P450 4A (Cyt P450; a non-peroxisomal clofibrate-inducible enzyme), the L-specific peroxisomal multifunctional protein (MFP1; a clofibrate-inducible enzyme), peroxisomal thiolase (thiol; a clofibrate-inducible enzyme), or catalase (cat; a peroxisomal enzyme not induced by clofibrate). The asterisk indicates the migration of a nonspecific, immunoreactive protein. (B, C) Primary hepatocytes from mouse embryos (13.5 days) of *Arf6*^+/+ ^and *Arf6*^-/- ^littermates from control and clofibrate-treated pregnant *Arf6*^+/- ^mice were isolated, seeded on collagen-coated cover glasses, cultured for 12 hours, and processed for indirect immunofluorescence microscopy. (B) Representative pictures showing that ARF6 ablation does not alter the localization of Pex14p, a peroxisomal membrane protein. The scale bar represents 10 μm. (C) The number of peroxisomes (per cell section; n > 15) is not substantially altered in wild-type and *Arf6*^-/- ^cells. The error bars indicate the standard deviation.

In order to investigate the potential role of Arf6 in peroxisome biogenesis, we examined the number of peroxisomes, peroxisomal morphology, and the localization of peroxisomal membrane and matrix proteins in *Arf6*^-/- ^fetal hepatocytes [31]. As it is well-known that peroxisomes in cultured hepatoma cells may exhibit distinct alterations of shape, size, and distribution dependent on culture conditions (including cell density, duration in culture, and the presence of specific growth factors) [32], we analyzed high, middle and low density hepatocyte populations from wild-type and knockout animals side-by-side. Probing these cells with antibodies raised against the peroxisomal membrane protein Pex14p (Figure 6C), the PTS1 protein catalase (see Additional file 3), the peroxisomal membrane protein PMP70 (data not shown) or the PTS2 protein thiolase (data not shown) showed that ablation of Arf6 did not visibly alter the localization of peroxisomal proteins in cultured fetal hepatocytes from both control and clofibrate-treated pregnant mice. Clofibrate is a hypolipidemic drug known to induce peroxisome proliferation in rodent liver (Figure 6A). In addition, no evidence could be found for an altered number (Figure 6B) or spatial distribution (see Figure 6C and Additional file 3) of peroxisomes. These findings may be surprising in light of our observations that the number of peroxisomes is altered in *S. cerevisiae *cells deficient in Arf3 (Figure 1C), the yeast orthologue of mammalian Arf6 [33]. We suggest that a functional redundancy of different Arf proteins may protect peroxisome biogenesis in mammals. In this context it is interesting to point out that every combination of double – but not single – knockdowns of Arf1, Arf3, Arf4 and Arf5 yielded a distinct pattern of defects in secretory and endocytic traffic in HeLa cells [34].

### Co-overexpression of Arf1 and Arf6 variants impairs peroxisomal protein import in mammalian cells

**Figure 7:**
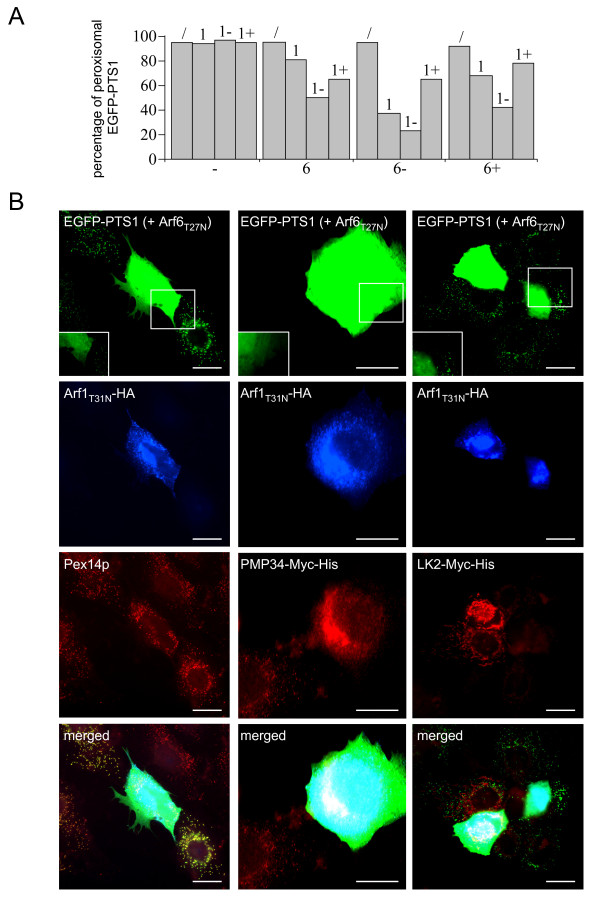
**Effect of co-overexpression of Arf1 and Arf6 variants on peroxisomal and mitochondrial protein import in PtK2 cells**. (A) Ptk2 cells were transiently transfected with plasmids coding for no protein (/), Arf1_WT_-HA (1), Arf1_T31N_-HA (1-), or Arf1_Q71L_-HA (1+) and/or a bicistronic plasmid encoding EGFP-PTS1 together with no other protein (-), non-tagged Arf6_WT _(6), Arf6_T27N _(6-), or Arf6_Q67L _(6+). After 36 hours, the cells were fixed and processed for fluorescence analysis. The subcellular localization of EGFP-PTS1 was determined by its punctate (peroxisomal) or diffuse staining pattern in at least 250 cells, and the results were quantified. (B) Representative images of the subcellular distribution pattern of EGFP-PTS1, HsPMP34-Myc-His, and HsLK2-Myc-His in Ptk2 cells co-overexpressing Arf1_T31N _and Arf6_T27N_. Note that the latter protein is encoded by the bicistronic expression vector coding for EGFP-PTS1, and a mislocalization of the reporter proteins is only observed in double-transfected cells. As (i) in mammalian cells the fluorescence intensity of EGFP-PTS1 is significantly higher upon mislocalization to the cytosol [77], and (ii) this increase may mask the (partial) association of this reporter protein with peroxisomes, insets are included in which the outlined regions are shown with moderately less intense green fluorescence signals. Scale bar: 20 μm.

As a previous study has shown that exogenously added Arf1 can bind to highly purified rat liver peroxisomes [19], we investigated whether or not overexpression of wild-type, GTP hydrolysis-defective or (dominant-negative) GTP binding-defective variants of Arf1 (Arf1_wt_, Arf1_Q71L _[35], and Arf1_T31N _[35], respectively) and Arf6 (Arf6_wt_, Arf6_Q67L _[36], and Arf6_T27N _[37], respectively), individually or in pairwise combinations, affected peroxisomal protein import in mammalian cells. These experiments demonstrated that overexpression of individual Arf variants had no effect on the localization of EGFP-PTS1, a peroxisomal matrix protein reporter (Figure 7A). However, when Arf1 and Arf6 variants were simultaneously expressed, all combinations yielded a mislocalization of the reporter protein to various degrees (Figure 7A). Interestingly, co-expression of the GDP-bound dominant-negative mutants Arf6_T27N _and Arf1_T31N _resulted in a partial to complete cytosolic localization of EGFP-PTS1 in approximately 80% of the cells (Figures 7A and 7B (left column and upper row)). Similar findings were observed for HsPMP34-Myc-His, a PMP reporter protein (Figure 7B, middle column). This observation indicates that the mislocalization of EGFP-PTS1 is most likely the indirect result of a peroxisomal membrane protein import deficiency. At first sight, this hypothesis seems to be at odds with the observation that a simultaneous expression of Arf6_T27N _and Arf1_T31N _did not have a visible effect on the subcellular localization of endogenous Pex14p (Figure 7, left column). However, as (i) the half-life of peroxisomes in cultured mammalian cells is approximately two days, and (ii) the turnover rate of Pex14p is rather low [38], the Pex14p signals observed in Figure 7B most likely represent pre-existing Pex14p molecules which were already localized to peroxisomes before Arf6_T27N _and Arf1_T31N _were co-overexpressed. Interestingly, upon closer inspection, we found that the staining pattern of Pex14p was slightly changed in at least some cells co-overexpressing Arf6_T27N _and Arf1_T31N _(see Additional file 4, compare the Pex14p signals in non- or single transfected cells with those in double-transfected cells). Indeed, a diffuse 'background' staining could be observed (most likely representing the pool of newly-synthesized Pex14p), and – upon a drastic enlargement of the images – it became also clear that the appearance of the Pex14p-immunoreactive particles was affected (see Additional file 5). The precise nature and mechanism of these changes remain to be determined. Importantly, Arf3, Arf4, and Arf5 could not substitute for Arf1 in these assays (data not shown). In addition, co-expression of the GDP-bound dominant-negative variants of Arf1 and Arf6 did not interfere with the sorting of HsLK2-Myc-His, a mitochondrial reporter protein (Figure 7B, right column).

## Discussion

In many instances, the formation of membrane-delimited compartments from pre-existing organelles is regulated by small GTPases of the Rab and Arf family [22,23,39,40]. A growing amount of evidence suggests that monomeric GTP-binding proteins may also play a role in peroxisome biogenesis. For example, mass spectrometry-based quantitative proteomic profiling studies have shown that in yeast Rho1 is recruited to peroxisomes upon their induction by oleate, and it has been suggested that this protein regulates the assembly state of actin on the peroxisome membrane, thereby controlling peroxisome membrane dynamics and biogenesis [41]. Recently, it has also been shown that MYA2, an isoform of *Arabidopsis thaliana *myosin XI, is targeted to peroxisomes through an interaction with AtRab2CA [42]. Furthermore, mammalian Arf1 and Arf6 have been shown to bind to isolated rat liver peroxisomes, and ScArf1 and ScArf3, the yeast orthologues of these proteins, have been implicated in the control of peroxisome proliferation [19]. A similar function has been suggested for *Trypanosoma brucei *Arf1, a protein which shares characteristics with both Arf1 and Arf6 and has a vital role in the maintenance of the endocytic pathway [43]. Interestingly, no Arf proteins have been identified in proteome studies of yeast and mammalian peroxisomes [41,44-50]. A possible explanation might be that the relative abundance of these proteins in the analyzed samples is extremely low.

In this study, we initially investigated whether or not yeast strains deficient in Arf1, Arf2, Arf3, Arl1, Cin4, Vps21, Ypt6, Ypt7, Ypt10, Ypt11, Ypt31, Ypt32, Ypt52 or Ypt53 were capable of growing on oleate as a sole carbon source and/or displayed a peroxisomal protein sorting defect. The results of these experiments demonstrated that all these strains, which embody every viable yeast deletion mutant of the Rab and Sar1/Arf subfamilies of small GTPases, contain functional peroxisomes. Interestingly, the average number of peroxisomes in oleate-grown cells was significantly upregulated in the Δ*arf1 *and Δ*arf3 *strains compared to the wild-type strain, and this phenotype was even more pronounced in the double Δ*arf1Δarf3 *deletion mutant. This observation indicates that ScArf1 and ScArf3 dampen peroxisome proliferation, either directly or indirectly. Although this result confirms previous observations that ScArf1 and ScArf3 are implicated in the control of peroxisome proliferation [19], it is not consistent with the authors' conclusion that ScArf1 is required for the oleate-induced peroxisome proliferation in *S. cerevisiae*. The reasons for the discrepancy between our results and the results previously presented might be several. For example, while we were making use of an *Arf1 *deletion mutant, Lay *et al*. [19] utilized an Arf1 temperature-sensitive mutant in an Δ*arf2 *background. Also, while these authors analyzed the peroxisome number per cell after 6 hours of growth on oleate, we analyzed the cells after 5 or 9 days of growth on oleate. Note that after transfer of the cells to oleate-containing medium, the number of peroxisomes per cell continuously increased as a function of time for at least 14 days, in the Δ*arf1 *and Δ*arf3 *strains compared to the wild-type strain (data not shown). At this point, it is not clear how Arf1 and Arf3 suppress oleate-induced peroxisome proliferation in *S. cerevisiae*. One possibility might be that these proteins are involved in the generation of peroxisome-derived vesicles, which transport ER-derived factors from peroxisomes back to the ER [7,8,12,16] or deliver metabolites, lipids, or (mistargeted) proteins to mitochondria [51]. Disturbances in such pathways would result in an increase in peroxisome number.

We next investigated whether or not endogenous Arf proteins were associated with the membrane of rat liver peroxisomes. In a first series of experiments, we obtained evidence that 1D9, a mouse monoclonal antibody with specificity for mammalian Arf1, Arf2, Arf3, Arf5, Arf6, and – to a lesser extent – Arf4 [30], recognized a 21 kDa band in highly purified peroxisomal membrane preparations. We could show that this 21 kDa protein was associated with the outside face of the peroxisomal membrane (as determined by immuno-electron microscopy), and identify this protein as Arf6 (as determined with Arf-specific antibodies). This protein, which is considered to be the mammalian orthologue of yeast Arf3, has been reported to act in a wide range of processes, including endocytosis, cytokinesis, phagocytosis, and the organization of the actin cytoskeleton [22]. Our observation that endogenous Arf6 is also associated with peroxisomes may seem surprising given that the subcellular localization of this protein has already been documented in several reports [30,52-54]. However, as (hepatic) peroxisomes occupy less than 2% of the cell volume [55], a partial (and transient) association of Arf6 with these organelles may have been easily missed. Also, as the strength of the signal and its detection depend heavily on the amount of protein and may be easily masked by stronger signals, the methods employed might not be sensitive enough. No evidence could be obtained for the association of any other endogenous Arf protein with the peroxisomal membrane. However, these results should be considered with caution as such proteins may dissociate during the purification procedure and be present on the peroxisomal membrane in concentrations below the detection limits of the employed Arf-specific antibodies.

To study the function of Arf6 in peroxisome biogenesis, we first examined the number of peroxisomes, peroxisomal morphology, and the location of (newly-synthesized) peroxisomal membrane and matrix proteins in (i) Ptk2 cells transiently overexpressing either wild-type Arf6 or a mutant defective in GTP-binding (Arf6_T27N_) or GTP-hydrolysis (Arf6_Q67L_) and (ii) *Arf6*^-/- ^fetal mouse hepatocytes [31]. Neither the overexpression of Arf6 variants nor the absence of Arf6 resulted in a detectable peroxisomal phenotype. Together, these observations suggest that peroxisome-bound Arf6 alone is not essential for peroxisome biogenesis in mammalian cells. The lack of a phenotypic defect is most probably the result of functional redundancy among individual Arf proteins [34], and since it has been reported that exogenously added Arf1 can bind to isolated rat liver peroxisomes [19], we decided to carry out another series of experiments in which Arf6_WT_, Arf6_T27N_, or Arf6_Q67L _were transiently co-overexpressed with either wild-type Arf1 or a mutant defective in GDP-binding (Arf1_T31N_,) or GTP-hydrolysis (Arf1_Q71L_). These experiments showed that a combined overexpression of Arf1 and Arf6 variants resulted in a (partial) mislocalization of newly-synthesized peroxisomal reporter proteins and an altered peroxisome morphology. Importantly, the intracellular transport pathway of HsLK2-Myc-His, a human mitochondrial multisubstrate lipid kinase [56], was not disturbed. Interestingly, combinations with Arf1_T31N _and Arf6_T27N _consistently displayed the strongest phenotypes. Similar observations were observed in CHO and HepG2 cells (data not shown). These mutants are thought to render endogenous Arf1 and Arf6 inactive, presumably by sequestering a select set of guanine nucleotide exchange factors [35,52]. Since a similar, but less drastic, phenotype was observed when other Arf6 and Arf1 variants were expressed, and Arf3, Arf4, and Arf5 could not substitute for Arf1, it is unlikely that the (partial) mislocalization of the peroxisomal reporter proteins is the result of indirect changes in the level of activation of other Arf subtypes. In addition, our observation that a similar phenotype was obtained with all Arf1 and Arf6 variants also suggests the necessity for a completion of the GDP/GTP cycle of these proteins to achieve their potential function in peroxisome biogenesis [[57], and references therein]. In summary, these findings – which suggest that Arf1 and Arf6 act in tandem to regulate a peroxisomal trafficking pathway – lend strong support to and extend the hypothesis that the cooperation of two Arf proteins at the same subcellular location is a general feature of Arf signaling [34].

To date, only one group has studied the potential relationship between ADP-ribosylation factors and peroxisome formation in mammalian cells, and shown that exogenously added Arf1 and Arf6 can bind to isolated rat liver peroxisomes [17,19]. In this work, we have extended these findings by showing that (i) also endogenous Arf6 associates with the cytoplasmic side of the peroxisomal membrane, and (ii) a combined overexpression of Arf6 and Arf1 variants selectively impairs peroxisomal protein import and alters peroxisome morphology. The molecular mechanisms underlying these phenotypic alterations remain to be clarified, since these Arf proteins do not interact directly with any of the currently identified human peroxins as tested with yeast- and bacterial two-hybrid assays (data not shown). Nevertheless, based on (i) a recent report showing that active Arf6 can recruit ARNO – a soluble Arf1 GTPase-activating guanine nucleotide exchange factor – to the plasma membrane [58], (ii) a growing body of evidence supporting the existence of vesicular trafficking pathways between peroxisomes and other subcellular compartments [8,51], and (iii) the observation that Arf1 is involved in the transport pathway of tomato bushy stunt virus 33 kDa replication protein from peroxisomes to the ER in *Nicotiana tabacum *cells [21], it is tempting to speculate that activated peroxisomal Arf6 is involved in the formation of peroxisome-derived vesicles by serving as an adaptor for the recruitment of not yet identified guanine nucleotide exchange factor(s) to sites at the peroxisomal membrane which in turn may lead to Arf1 activation and cytosolic coat protein recruitment. Depending on the functional redundancy among the Arf proteins and the role of the peroxisome-derived vesicles in different organisms, the inability of a cell to form such vesicles may result in an increase in peroxisome number or a block in the formation of new organelles.

## Conclusion

In this study, we provide evidence that endogenous Arf6, the mammalian orthologue of *S. cerevisiae *Arf3, can tightly associate with the outside face of the peroxisomal membrane. We also demonstrated that a combined overexpression of Arf6 and Arf1 variants caused mislocalization of newly-synthesized peroxisomal proteins and resulted in an alteration of peroxisome morphology. These observations suggest that Arf6 – albeit not essential – is a key player in mammalian peroxisome biogenesis. In addition, these findings extend the concept that specific Arf isoform pairs may act in tandem to regulate exclusive trafficking pathways. The precise mechanism by which Arf6 functions in peroxisome biogenesis remains to be determined.

## Methods

### Animals

Male Wistar rats (weighing 200–250 g), Swiss-Webster mice, and New Zealand White rabbits were kept in a constant light-dark cycle on a standard laboratory diet. The rats were fasted 16 hours before sacrifice. *Arf6*^+/- ^C57BL/6 mice were maintained in a pathogen-free facility as described elsewhere [31]. To study the effect of a peroxisome proliferator on the numerical abundance, structure, and protein import competence of peroxisomes in *Arf6*^-/- ^fetal hepatocytes, pregnant *Arf6*^+/- ^mice were fed a diet containing 0.3% (w/w) clofibrate beginning at day 6.5 of gestation [59]. All animal experiments were approved by the local institutional ethics committees.

### Plasmids

Cloning vectors were obtained from Qiagen (pQE30), Clontech (pEGFP-N1, pIRES2-EGFP) or Stratagene (pCMV-Tag4A, B), and the oligonucleotides synthesized for this study were from Eurogentec. PCR applications were performed routinely using *Pfx *DNA polymerase (Invitrogen). Restriction enzymes were purchased from TaKaRa. The *Escherichia coli *strain *Top10F' *(Invitrogen) was used for all DNA manipulations. Full-length cDNAs coding for human (*Homo sapiens*, Hs) Arf1 (accession number NM_001649), Arf3 (accession number NM_001659), Arf4 (accession number NM_001660), Arf5 (accession number NM_001662), and Arf6 (accession number NM_001663) were obtained from the I.M.A.G.E. consortium (clones 3463523, 2967578, 6157009, 6720876, and 5212700, respectively) [60]. The mammalian expression vectors coding for *Bos Taurus *(Bt) Arf1_WT_-HA, HsArf1_T31N_-HA, or HsArf1_Q71L_-HA [61] were obtained from Addgene (plasmids 10830, 10833, and 10832, respectively). The plasmids coding for HsArf6-HA, HsArf6_T27N_-HA, or HsArf6_Q67L_-HA were kindly provided by Dr. P. Chavrier (Institut Curie, Paris, France). The yeast expression vector encoding EGFP-PTS1 (pJR233) was kindly provided by Dr. A. Hartig (University of Vienna, Austria). The mammalian expression plasmids pTW360 (coding for HsPMP34-Myc-His) and pSG005 (coding for HsLK2-Myc-His) have been described elsewhere [56,62]. The bicistronic expression plasmids coding for EGFP-PTS1 and non-tagged HsArf1_T31N _(pEA42), HsArf6_WT _(pEA38) or HsArf6_Q67L _(pEA91) were constructed by amplifying the corresponding cDNAs by PCR (Arf1 primers: 5'-gtttgagatctcatggggaacatcttcgcc-3' (HsArf1F1) and 5'-caaaagtcgactcacttctggttccggagc-3' (HsArfR4); Arf6 primers: 5'-gtttgagatctgatggggaaggtgctatcc-3' (HsArf6f1) and 5'-gtgtggtcgactcattaagatttgtagttagagg-3' (HsArf6r4)), and cloning the *Bgl *II/*Sal *I-digested PCR products into the multiple cloning site of pIRES2-EGFP-PTS1 (pMF891) [63] digested with the same restriction enzymes. The bicistronic plasmid coding for EGFP-PTS1 and non-tagged HsArf6_T27N _(pEA43) was constructed by fusion PCR. In a first PCR reaction, two PCR fragments (template: pEA38; primers: 5'-ggactttcctacttggcag-3' (pIRES2-EGFP-f1) and 5'-ggattgtattcttgccggccgcgtcc-3' (HsArf6T27Nr1) (fragment 1), 5'-ccggcaagaatacaatcctgtacaagttgaag-3' (HsArf6T27Nf1) and HsArf6r4 (fragment 2)) were generated. These PCR fragments were fused and used as a template in a second PCR reaction (primers: HsArf6f1, HsArf6r4). After digestion with *Bgl *II and *Sal *I, the fusion fragment was subcloned into the *Bgl *II/*Sal *I-digested pIRES2-EGFP-PTS1 vector. The mammalian expression vectors coding for HsArf3-FLAG (pMF1599), HsArf4-FLAG (pMF1598) or Arf5-FLAG (pMF1577) were constructed by transferring the *Bam *HI/*Bgl II*-digested (pMF1577, pMF1599) or *Bgl *II/*Sal *I-digested (pMF1598) cDNA inserts from pEA51, pEA35, and pEA52 into the *Bam HI*-digested pCMV-Tag4A (pMF1577), *Bgl *II/*Sal *I-digested pCMV-Tag4B (pMF1598), or *Bgl *II/*Bam *HI-digested pCMV-Tag4B (pMF1599) vectors. To generate pMF214, a bacterial expression plasmid coding for (His)_6_-HsPex3p_(229–364) _[64], the 403 bp *Bam *HI – *Hind *III fragment of the *PEX3 *open reading frame was ligated into *Bam *HI/*Hind *III-restricted pQE30. All essential constructs were verified by DNA sequencing (Agowa).

### Yeast strains

The haploid *Saccharomyces cerevisiae *strains BY4741 (genotype: *MATa*; *his3Δ1*; *leu2Δ0*; *met15Δ0*; *ura3Δ0*; accession number Y00000) and BY4742 (genotype: *MATα *; *his3Δ1*; *leu2Δ0*; *lys2Δ0*; *ura3Δ0*; accession number Y10000) as well as the corresponding null mutants (open reading frame::KanMX4) of *arf1 *(Y03890), *arf2 *(Y03835), *arf3 *(Y01870 and Y11870), *arl1 *(Y03304), *cin4 *(Y06744), *pex5 *(Y03603), *vps21 *(Y01865), *ypt6 *(Y05171), *ypt7 *(Y00575), *ypt10 *(Y03404), *ypt11 *(Y01140), *ypt31 *(Y06583), *ypt32 *(Y04576), *ypt52 *(Y05085), and *ypt53 *(Y07232) were obtained from Euroscarf. Yeast cells were transformed and selected as described [65]. In order to construct the double *arf1arf3 *deletion strain, the single *arf1 *(Y03890) and *arf3 *(Y11870) deletion strains were crossed with each other. Diploids were selected on -lys -met medium and checked by mating type PCR. The diploids were sporulated and subjected to tetrad analysis. Double deletion strains were selected on medium containing Geneticin (2:2 segregation for growth on Geneticin indicates that the two mutations are together) and verified by PCR analysis. To assay yeast cells for growth in the presence of oleate as a sole carbon source, the cells were pelleted, washed, and resuspended in sterile water to an optical density of 0.4 at 600 nm. Ten-fold serial dilutions were spotted on oleic acid plates containing 0.1% (v/v) oleic acid, 0.4% (v/v) Tween-40, 0.1% (w/v) yeast extract (Difco), 2% (w/v) agar, and synthetic dropout medium [66].

### Antibodies

The polyclonal antiserum against (His)_6_-HsPex3p_(229–364) _was raised in New Zealand White rabbits as previously described [67]. The mouse polyclonal antiserum against bovine catalase and the rabbit polyclonal antisera against HsPex13p, HsPex14p, HsPex19p, rat peroxisomal thiolase, and rat liver peroxisomal matrix proteins (ab-MF16; used to detect catalase and urate oxidase on Western blot) are described elsewhere [63,64,68,69]. The 1-9E10.2 hybridoma cell line producing anti-Myc antibody [70] was obtained from A.T.C.C. All other primary antibodies were commercially obtained. This list includes (i) rabbit polyclonal antibodies against Arf (Santa Cruz Biotechnology, sc-9063), glutamate dehydrogenase (Rockland Immunochemicals, 100–4158), PMP70 (Zymed Laboratories, 71–8300), HA (GeneTex, GTX29110), cytochrome P450 4A (Affinity BioReagents, PA3-033), and LAMP2 (Sigma, L 0068), (ii) an affinity-purified goat polyclonal antibody against Arf (Santa Cruz Biotechnology, sc-7622), (iii) a rabbit monoclonal antibody against Arf1 (Abcam, ab32524, clone [EP442Y]), and (iv) mouse monoclonal antibodies against Arf3 (BD Biosciences, 610785, clone [41]), nucleoporin 60 (BD Biosciences, N43620), Bip/GRP78 (BD Biosciences, G73320), pan-cadherin (Sigma, C 1821), Golgi 58 K (Sigma, G 2404), Arf (Abcam, ab2806, clone [1D9]), Arf5 (Abnova, H00003428-M05, clone [1B4]), Arf6 (Santa Cruz Biotechnology, sc-7971, clone [3A-1]), and HA (Roche, 11583816001, clone [12CA5]). The goat anti-rabbit and anti-mouse IgGs conjugated to 12 nm and 18 nm colloidal gold particles, respectively, were obtained from Jackson ImmunoResearch Laboratories. All other secondary antibodies were from Sigma.

### Cell culture, transfections, and (immuno)fluorescence microscopy

Primary hepatocytes from mouse embryos (13.5 days) of *Arf6*^+/+ ^and *Arf6*^-/- ^littermates were isolated and cultivated as described [31]. After 12 hours, the cells were fixed with ice-cold paraformaldehyde (4% (w/v) in PBS), washed with PBS, and stored until needed. *Potorous tridactylis *(kangaroo rat) kidney (Ptk2) cells (a gift from Dr. M. Mareel, University Hospital of Ghent, Belgium), allowing for easier imaging given their flat profile [71], were cultured in DMEM/F-12 medium supplemented with 10% (v/v) fetal calf serum, 2 mM Glutamax (Invitrogen), an antibiotic-antimycotic (100 μg/ml penicillin G, 100 μg/ml streptomycin sulphate, 0.25 μg/ml amphotericin B) mixture (Invitrogen), and 5 μg/ml Plasmocin (Amaxa) in a humidified 37°C, 5% CO_2 _incubator, and transiently transfected by using Lipofectamine Plus (Invitrogen). The cells were fixed with paraformaldehyde (see above) 36 hours post-transfection. Processing of the cell samples for (immuno)fluorescence microscopy was carried out as described [72], and fluorescence was evaluated on a CellM imaging station (Olympus) equipped with U-MNUA2, U-MNIBA3, and U-MWIY2 fluorescence mirror units. The Olympus image analysis and particle detection software was used to determine the peroxisome density in *Arf6*^+/+ ^and *Arf6*^-/- ^cells.

### Preparation of subcellular fractions and isolation of peroxisomal membranes

Rat liver was homogenized in 0.25 M sucrose, 5 mM MOPS (pH 7.2), 1 mM EDTA (pH 7.2), 0.1% (v/v) ethanol, 1 mM dithiothreitol (DTT), and proteinase inhibitor (PI) mixture (1 μg/ml aprotinin, 0.5 μg/ml leupeptin, 1 μg/ml α2-macroglobulin, and 1 μg/ml chymostatin) and fractionated by differential centrifugation into a nuclear, heavy mitochondrial, light mitochondrial, microsomal, and cytosolic fraction as described before [73]. Peroxisomes were purified from the light mitochondrial fraction by (i) isopycnic centrifugation in an iso-osmotic self-generating Percoll gradient [74], (ii) centrifugation in a Nycodenz step gradient [75], or (iii) centrifugation of peroxisome-enriched fractions from the Percoll gradient in a Nycodenz step gradient [73]. In order to isolate peroxisomal membranes, peroxisomal peak fractions in the Nycodenz gradient were subjected twice to sonication in 10 mM pyrophosphate buffer (pH 9.0) and centrifuged at 100,000 × g for 1 hour [74]. To increase the purity of the peroxisomal membranes, the membrane pellets were resuspended in 1 ml of sucrose 52% (w/v) in 0.1 M sodium carbonate (pH 11.5) and floated in an alkaline linear sucrose gradient essentially as described previously [76], with minor modifications. Briefly, resuspended membrane pellets were carefully layered under 4 ml of a continuous gradient (ranging from 12% to 44% (w/v) sucrose in 0.1 M sodium carbonate (pH 11.5)). In order to avoid the pelleting of protein aggregates at the bottom of the centrifugation tubes, a 100 μl cushion of sucrose 80% (w/v) in 0.1 M sodium carbonate was layered under the resuspended membrane pellets. The sucrose gradient was centrifuged at 170,000 × g for 18 hours in a SW55Ti rotor (Beckman Instruments) and fractionated into 200–250 μl aliquots. The proteins present in each fraction were precipitated with trichloroacetic acid and deoxycholate, and processed for SDS-PAGE and immunoblotting.

### Immunoelectron microscopy

Percoll, Nycodenz and Percoll/Nycodenz gradient fractions enriched with peroxisomes were fixed in a freshly prepared solution containing 0.25% (v/v) glutaraldehyde-containing, 5 mM MOPS (pH 7.2), 1 mM EDTA (pH 7.2), 1 mM DTT, 0.1% (v/v) ethanol, and PI mixture. In order to prevent osmotic rupture of the organelles, the fixative was supplemented with 0.25 M sucrose (Percoll fractions) or Nycodenz (final density: 1.20 g/ml) (Nycodenz and Percoll/Nycodenz fractions). Fixed samples containing 75 μg or 150 μg of protein were pelleted (11,000 × g, 2 min) and washed by resuspension and pelleting in (i) homogenization buffer (see above), (ii) TBS buffer (0.1 M Tris-HCl (pH 7.4), 9% (w/v) NaCl), and (iii) TBS buffer supplemented with 0.1% (w/v) glycine. Non-specific protein binding sites were blocked for 45 min in TBS buffer supplemented with 1% (w/v) BSA, and the organelle preparations were subsequently incubated with the primary antibodies (mouse 1D9 (1:10 – 1:500) or mouse anti-Arf6 (1:50 – 1:100) and rabbit anti-Pex14p (1:20 – 1:1000) or rabbit anti-PMP70 (1:20 – 1:1000) diluted in TBST (TBS buffer supplemented with 0.05% (v/v) Tween-20)) overnight at 4°C. The next morning, the preparations were washed three times with TBS buffer, incubated with the secondary antibodies (goat anti-rabbit (1:5 – 1:20) and anti-mouse IgGs (1:5 – 1:10) conjugated to 12 nm and 18 nm colloidal gold particles, respectively, diluted in TBST) for two hours at 4°C, and washed three times with TBS buffer (and pelleted for 2 min at decreasing pelleting speeds: 11,000 × g, 5,000 × g, and 2,000 × g). Negative controls were processed in parallel by addition of TBST buffer instead of the primary antibodies. Hereafter, the immuno-treated organelle samples were fixed in TBS buffer supplemented with 0.1 M cacodylate (pH 7.4) and 1% (v/v) glutaraldehyde for 30 min, and subsequently washed with 0.1 M cacodylate (pH 7.4). Afterwards, the samples were post-fixed with aqueous potassium ferricyanide-reduced osmium tetroxide (final concentration: 1% (w/v) OsO_4_; 150 mg/ml K_4 _[Fe(CN)_6_]) for 45 min, shortly rinsed with Milli-Q water, and immobilized on Formvar-coated 400 mesh nickel grids (see below) or embedded in tepid agar-agar (7.5% (w/v) in Milli-Q). The in agar-agar-embedded pellets were carefully cut into small blocks and dehydrated by passage through graded series of ethanol (70% (v/v), 80% (v/v), 90% (v/v), and absolute), transferred to propylene oxide, and embedded in Epoxy resin (Agar 100 Resin Kit, Agar Scientific). The Epon blocks were trimmed and ultrathin sections (75 nm and 300 nm) were put on a 200 mesh copper/rhodium grid. The organelles were contrasted with 2% (w/v) uranyl acetate and Reynold's lead citrate for 2 min and 40 sec, respectively, and rinsed with Milli-Q water. To immobilize immunostained or non-immunostained glutaraldehyde-fixed organelle samples directly on Formvar-coated 400 mesh nickel grids, the grids were first incubated with a poly-L-lysine solution (0.01% (w/v) poly-L-lysine, 0.625% (w/v) boric acid, 0.955% (w/v) sodium tetraborate) for 45 min and subsequently incubated on a drop of the organelle samples for another 45 min. Next, the grids containing immunostained samples were put on a drop of TBS for 10 min, shortly rinsed with the same buffer, and dried. The grids containing non-immunostained organelle samples were washed for 15 min on a drop of TBS and 5 min on a drop of TBS supplemented with 0.1% (w/v) glycine, blocked for 45 min on a drop TBS supplemented with 4% (w/v) BSA, and for one hour incubated with primary and secondary antibodies diluted in TBST supplemented with 0.1% (w/v) BSA. Each antibody incubation was ended by washing the immobilized organelles 12 times for 5 min on a drop of TBS. Negative controls were processed in parallel by addition of TBST buffer instead of the primary antibodies. Finally, the organelle samples were shortly rinsed with TBS, negatively stained by incubating them for 3 min with 0.25% (w/v) uranyl acetate, and dried on a filter paper. All samples were examined with a LEO 906 electron microscope (Zeiss).

### Statistics

Statistical analyses were performed with SAS (Statistical Analysis System) software. ANOVA (Analysis of Variance) was used to determine the differences among independent groups of numerical values, and multiple comparisons with Tukey-Kramer adjustment were used to determine which groups were significantly different from the wild-type group. The significance level was chosen to be 0.05.

## Authors' contributions

EAA carried out the phenotypic analysis of the yeast strains, performed the immunofluorescence microscopic experiments, participated in the preparation and analysis of the subcellular fractions and the electron microscopy samples, and helped to draft the manuscript. CB prepared the expression constructs and performed the yeast transformations. EB supervised the electron microscopy experiments. TH and YK provided the primary hepatocytes from mouse embryos of *Arf6*^+/+ ^and *Arf6*^-/- ^littermates from control and clofibrate-treated pregnant *Arf6*^+/- ^mice. PVD constructed the Δ*arf1Δarf3 *double deletion strain. SJH helped to analyze the immunofluorescence microscopy samples. GM, PVV and MF initiated the work hypothesis and participated in the study design. MF and PVV supervised the work. MF coordinated the study and drafted the manuscript. All authors read and approved the final manuscript.

## Supplementary Material

Additional file 1 **Phenotypic analysis of the oleate-grown Δ*arf1Δarf3 S. cerevisiae *strain**. Serial dilutions of wild-type (WT) yeast cells (strain BY4741) and yeast cells deficient in Arf1 (Δ*arf1*), Arf3 (Δ*arf3*), or Arf1 and Arf3 (Δ*arf1Δarf3*) expressing EGFP-PTS1 were spotted onto plates with oleate as a sole carbon source. The plates were subsequently incubated at 30°C for five days. (A) Oleate consumption was scored by halo formation. (B) The subcellular distribution pattern of EGFP-PTS1 was visualized by fluorescence microscopy. The scale bar represents 5 μm. (C) The number of peroxisomes per cell was counted in randomly selected cells. The mean number of peroxisomes per cell is indicated by an asterisk. At least 150 oleate-grown cells were scored. Click here for file

Additional file 2 **Specificity of the monoclonal anti-Arf6 antibody**. (A) Multiple sequence alignment of the rat Arf protein sequences. Amino acids identical in all aligned sequences are shown in red. Amino acid residues identical in five out of six sequences are shown in blue. Note that (i) Arf1 and Arf2 share the highest sequence identity (96% identical at the amino acid level; different amino acids are shaded in green), (ii) the latter protein is absent in humans, (iii) the amino acid sequences of Arf1, Arf3, Arf5 and Arf6 are completely identical in rat and human, and (iv) the amino acids not identical in rat and human Arf4 are highlighted in yellow. (B) Immunoblot analysis of equal amounts of extracts from CHO cells transfected with a monocistronic plasmid coding for Arf4-EGFP or a bicistronic plasmid encoding EGFP-PTS1 and no protein (-) or non-tagged human Arf1, Arf3, Arf5, or Arf6 proteins. The blots were probed with antibodies against EGFP (α-EGFP) or Arf6 (α-Arf6). Note that the expression levels of EGFP-PTS1 allow the indirect quantification of the Arf expression levels. The arrows indicate the migration of the full-length proteins. The arrowheads mark the Arf4-EGFP degradation products. The migration of relevant molecular mass markers (expressed in kDa) is shown at the left. (C) Immunoblot analysis of equal amounts of extracts from bacteria expressing (His)_6_-GST (H_6_-GST)-tagged human Arf proteins or a negative control protein (H_6_-GST-DCOH). The blots were probed with antibodies against (His)_6 _(α-H_6_) or Arf6 (α-Arf6). Note that, as – based on a Ponceau S staining – the expression levels of the H_6_-GST-tagged proteins varied greatly, the blots were cut into three pieces (each containing two conditions yielding similar amounts of recombinant protein) and incubated for different times in alkaline phosphatase-NBT/BCIP staining solution in order to normalize the signal intensities for equal amounts of recombinant protein. The arrows mark full-length proteins, the arrowheads point to degradation products. Click here for file

Additional file 3 **Arf6 ablation does not alter the localization of catalase in fetal mouse hepatocytes**. Primary hepatocytes from mouse embryos (13.5 days) of *Arf6*^+/+ ^and *Arf6*^-/- ^littermates from control (-CF) and clofibrate-treated (+CF) pregnant *Arf6*^+/- ^mice were isolated, seeded on collagen-coated cover glasses, cultured for 12 hours, and processed for indirect immunofluorescence microscopy with antibodies specific for catalase, a peroxisomal matrix protein. Scale bar: 20 μm. Click here for file

Additional file 4 **Effect of co-overexpression of Arf1**_T31N _**and Arf6**_T27N _**on peroxisomal protein import in Ptk2 cells**. Ptk2 cells were transiently transfected with a plasmid coding for Arf1_T31N_-HA and a bicistronic plasmid encoding EGFP-PTS1 together with Arf6_T27N_. After 36 hours, the cells were fixed and processed for fluorescence analysis. The top row shows three merged images of the signals observed for Arf1_T31N_-HA (blue), EGFP-PTS1 (green), and endogenous Pex14p (red). The other rows represent enlarged views of the individual colour components of the areas shown in the insets. Note that the simultaneous expression of Arf6_T27N _(encoded by the same plasmid as EGFP-PTS1) and Arf1_T31N _has a strong influence on the localization of newly-synthesized EGFP-PTS1, but only a minor effect on the localization of endogenous Pex14p. Possible explanations for this apparent discrepancy are reviewed in the Results section of the main manuscript. Scale bar: 20 μm. Click here for file

Additional file 5 **Effect of co-overexpression of Arf1**_T31N _**and Arf6**_T27N _**on the appearance of Pex14p-immunoreactive particles in Ptk2 cells**. Ptk2 cells were transiently transfected with a plasmid coding for Arf1_T31N_-HA and a bicistronic plasmid encoding EGFP-PTS1 together with Arf6_T27N_. After 36 hours, the cells were fixed and processed for fluorescence analysis. The top row shows three images of the signals observed for endogenous Pex14p (see Additional file 4, lower panels). The insets show an enlargement of the outlined regions. +, cell co-overexpressing Arf1_T31N _and Arf6_T27N_; -, cell overexpressing only Arf6_T27N_; 0, non-transfected cell (for overview images, see Additional file 4). Scale bar: 20 μm. Click here for file
