# Supplementary figures and images for: Small G proteins in peroxisome biogenesis: the potential involvement of ADP-ribosylation factor 6
Source: BMC Cell Biol. 2009 Aug 17;10:58. doi: 10.1186/1471-2121-10-58 (PMC3224584; doi:10.1186/1471-2121-10-58)

A

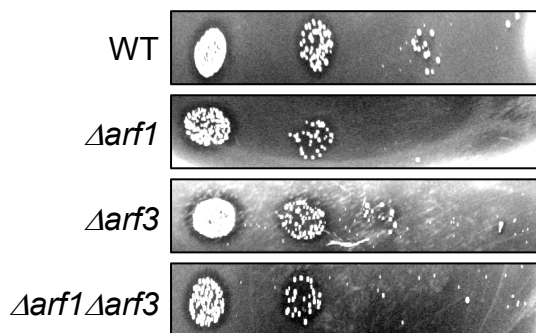

B

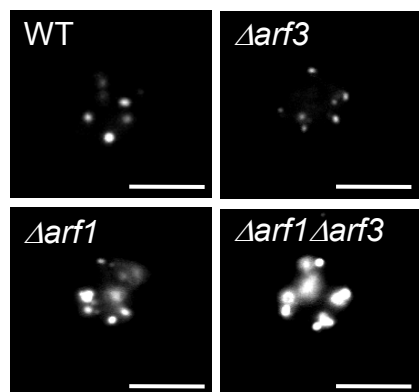

C

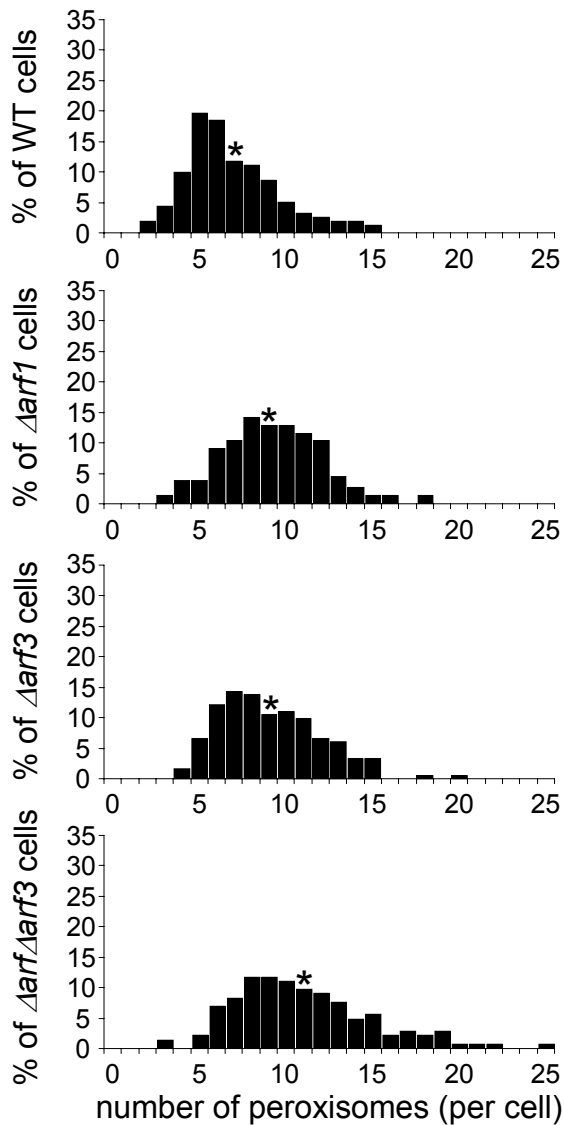

Supplement: Additional file 1 — Phenotypic analysis of the oleate-grown Δarf1Δarf3 S. cerevisiae strain. Serial dilutions of wild-type (WT) yeast cells (strain BY4741) and yeast cells deficient in Arf1 (Δarf1), Arf3 (Δarf3), or Arf1 and Arf3 (Δarf1Δarf3) expressing EGFP-PTS1 were spotted onto plates with oleate as a sole carbon source. The plates were subsequently incubated at 30°C for five days. (A) Oleate consumption was scored by halo formation. (B) The subcellular distribution pattern of EGFP-PTS1 was visualized by fluorescence microscopy. The scale bar represents 5 μm. (C) The number of peroxisomes per cell was counted in randomly selected cells. The mean number of peroxisomes per cell is indicated by an asterisk. At least 150 oleate-grown cells were scored. [file 1471-2121-10-58-S1.pdf]

*Arf6*<sup>+/+</sup> (E13.5)

*Arf6*<sup>-/-</sup> (E13.5)

- CF

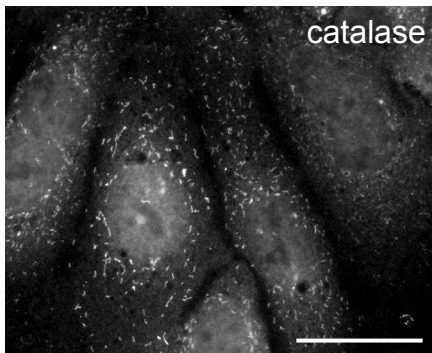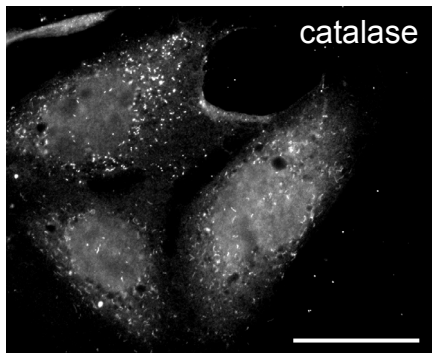

+ CF

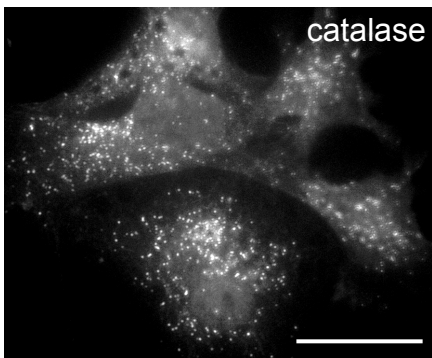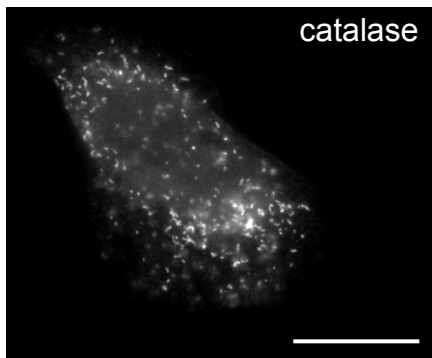

Supplement: Additional file 3 — Arf6 ablation does not alter the localization of catalase in fetal mouse hepatocytes. Primary hepatocytes from mouse embryos (13.5 days) of Arf6+/+ and Arf6-/- littermates from control (-CF) and clofibrate-treated (+CF) pregnant Arf6+/- mice were isolated, seeded on collagen-coated cover glasses, cultured for 12 hours, and processed for indirect immunofluorescence microscopy with antibodies specific for catalase, a peroxisomal matrix protein. Scale bar: 20 μm. [file 1471-2121-10-58-S3.pdf]

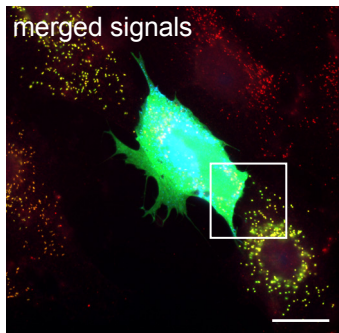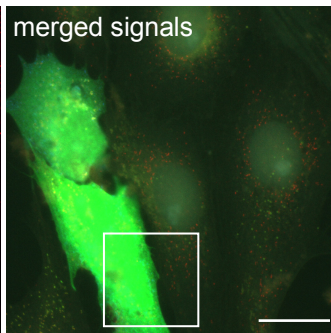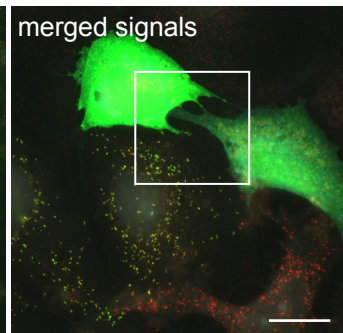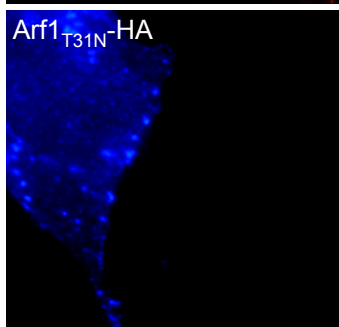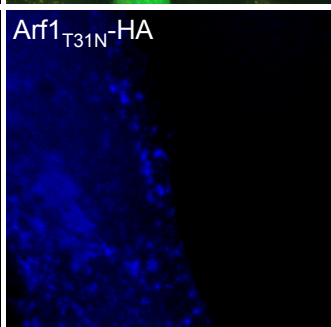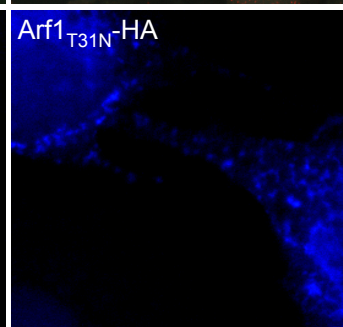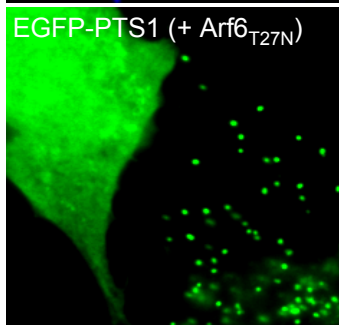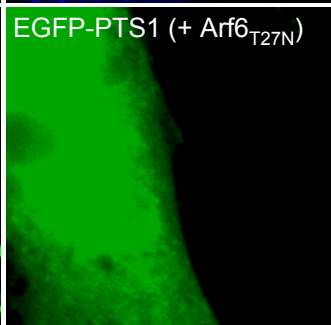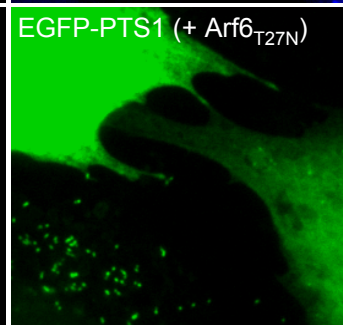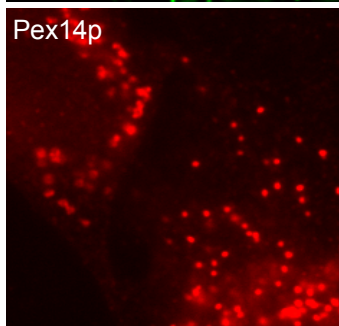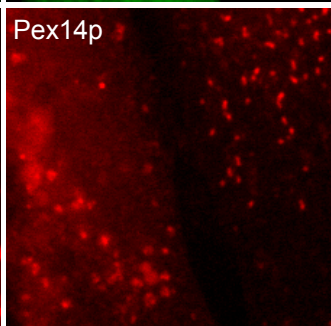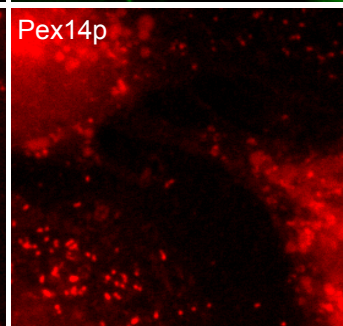

Supplement: Additional file 4 — Effect of co-overexpression of Arf1T31N and Arf6T27N on peroxisomal protein import in Ptk2 cells. Ptk2 cells were transiently transfected with a plasmid coding for Arf1T31N-HA and a bicistronic plasmid encoding EGFP-PTS1 together with Arf6T27N. After 36 hours, the cells were fixed and processed for fluorescence analysis. The top row shows three merged images of the signals observed for Arf1T31N-HA (blue), EGFP-PTS1 (green), and endogenous Pex14p (red). The other rows represent enlarged views of the individual colour components of the areas shown in the insets. Note that the simultaneous expression of Arf6T27N (encoded by the same plasmid as EGFP-PTS1) and Arf1T31N has a strong influence on the localization of newly-synthesized EGFP-PTS1, but only a minor effect on the localization of endogenous Pex14p. Possible explanations for this apparent discrepancy are reviewed in the Results section of the main manuscript. Scale bar: 20 μm. [file 1471-2121-10-58-S4.pdf]

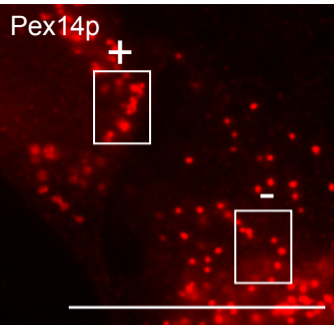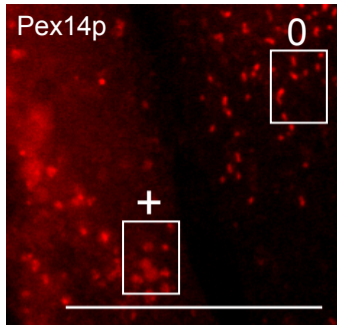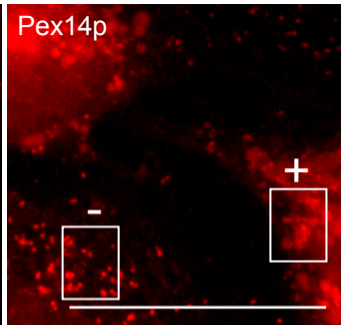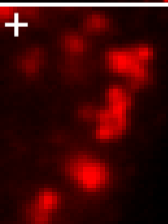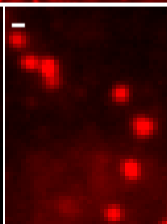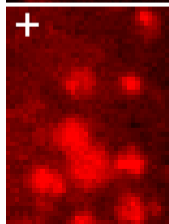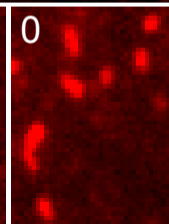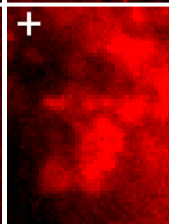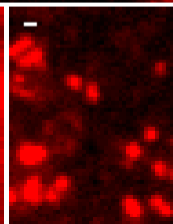

Supplement: Additional file 5 — Effect of co-overexpression of Arf1T31N and Arf6T27N on the appearance of Pex14p-immunoreactive particles in Ptk2 cells. Ptk2 cells were transiently transfected with a plasmid coding for Arf1T31N-HA and a bicistronic plasmid encoding EGFP-PTS1 together with Arf6T27N. After 36 hours, the cells were fixed and processed for fluorescence analysis. The top row shows three images of the signals observed for endogenous Pex14p (see Additional file 4, lower panels). The insets show an enlargement of the outlined regions. +, cell co-overexpressing Arf1T31N and Arf6T27N; -, cell overexpressing only Arf6T27N; 0, non-transfected cell (for overview images, see Additional file 4). Scale bar: 20 μm. [file 1471-2121-10-58-S5.pdf]
