# Supplementary material for: Small G proteins in peroxisome biogenesis: the potential involvement of ADP-ribosylation factor 6
Source: BMC Cell Biol. 2009 Aug 17;10:58. doi: 10.1186/1471-2121-10-58 (PMC3224584; doi:10.1186/1471-2121-10-58)
Supplement: Additional file 2 — Specificity of the monoclonal anti-Arf6 antibody. (A) Multiple sequence alignment of the rat Arf protein sequences. Amino acids identical in all aligned sequences are shown in red. Amino acid residues identical in five out of six sequences are shown in blue. Note that (i) Arf1 and Arf2 share the highest sequence identity (96% identical at the amino acid level; different amino acids are shaded in green), (ii) the latter protein is absent in humans, (iii) the amino acid sequences of Arf1, Arf3, Arf5 and Arf6 are completely identical in rat and human, and (iv) the amino acids not identical in rat and human Arf4 are highlighted in yellow. (B) Immunoblot analysis of equal amounts of extracts from CHO cells transfected with a monocistronic plasmid coding for Arf4-EGFP or a bicistronic plasmid encoding EGFP-PTS1 and no protein (-) or non-tagged human Arf1, Arf3, Arf5, or Arf6 proteins. The blots were probed with antibodies against EGFP (α-EGFP) or Arf6 (α-Arf6). Note that the expression levels of EGFP-PTS1 allow the indirect quantification of the Arf expression levels. The arrows indicate the migration of the full-length proteins. The arrowheads mark the Arf4-EGFP degradation products. The migration of relevant molecular mass markers (expressed in kDa) is shown at the left. (C) Immunoblot analysis of equal amounts of extracts from bacteria expressing (His)6-GST (H6-GST)-tagged human Arf proteins or a negative control protein (H6-GST-DCOH). The blots were probed with antibodies against (His)6 (α-H6) or Arf6 (α-Arf6). Note that, as – based on a Ponceau S staining – the expression levels of the H6-GST-tagged proteins varied greatly, the blots were cut into three pieces (each containing two conditions yielding similar amounts of recombinant protein) and incubated for different times in alkaline phosphatase-NBT/BCIP staining solution in order to normalize the signal intensities for equal amounts of recombinant protein. The arrows mark full-length proteins, the arrowheads point [file 1471-2121-10-58-S2.pdf]

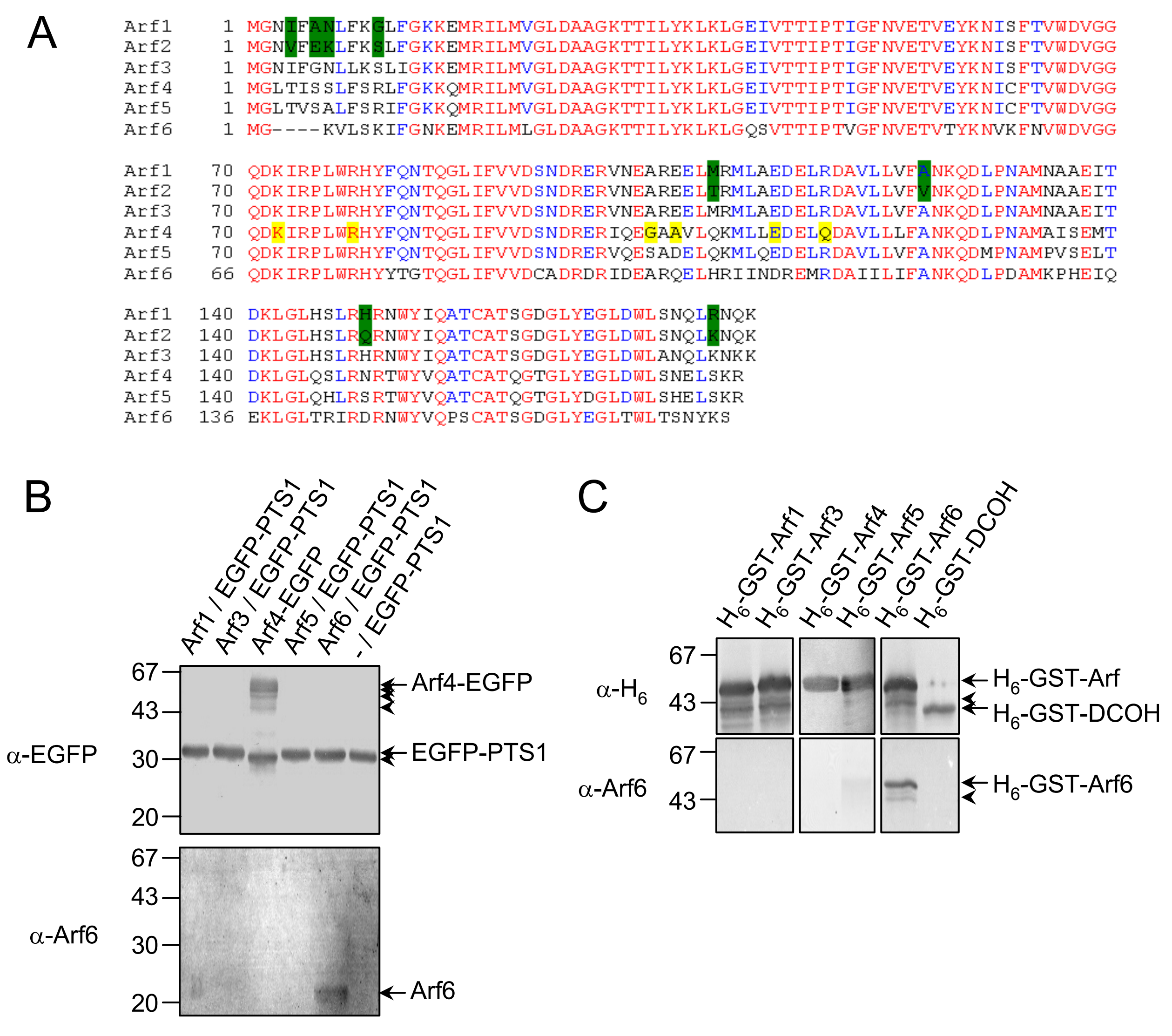
A
Arf1 1 MGNIFANLFKGLFGKKEMRILMVGLDAAGKTTILYKLKLGEIVTTIPTIGFNVETVEYKNISFTVWDVGG
Arf2 1 MGNVFEKLFKSLFGKKEMRILMVGLDAAGKTTILYKLKLGEIVTTIPTIGFNVETVEYKNISFTVWDVGG
Arf3 1 MGNIFGNLLKSLIGKKEMRILMVGLDAAGKTTILYKLKLGEIVTTIPTIGFNVETVEYKNISFTVWDVGG
Arf4 1 MGLTISSLFSRLFGKKQMRILMVGLDAAGKTTILYKLKLGEIVTTIPTIGFNVETVEYKNICFTVWDVGG
Arf5 1 MGLTVSALFSRIFGKKQMRILMVGLDAAGKTTILYKLKLGEIVTTIPTIGFNVETVEYKNICFTVWDVGG
Arf6 1 MG----KVLSKIFGNKEMRILMLGLDAAGKTTILYKLKLGQSVTTIPTVGFNVETVTYKNVKFNVWDVGG
Arf1 70 QDKIRPLWRHYFQNTQGLIFVVDSNDRERVNEAREELMRMLAEDELRDAVLLVFANKQDLPNAMNAAEIT
Arf2 70 QDKIRPLWRHYFQNTQGLIFVVDSNDRERVNEAREELTRMLAEDELRDAVLLVFVNKQDLPNAMNAAEIT
Arf3 70 QDKIRPLWRHYFQNTQGLIFVVDSNDRERVNEAREELMRMLAEDELRDAVLLVFANKQDLPNAMNAAEIT
Arf4 70 QDKIRPLWRHYFQNTQGLIFVVDSNDRERIQEGAAVLQKMLLEDELQDAVLLLFANKQDLPNAMAISEMT
Arf5 70 QDKIRPLWRHYFQNTQGLIFVVDSNDRERVQESADELQKMLQEDELRDAVLLVFANKQDMPNAMPVSELT
Arf6 66 QDKIRPLWRHYYTGTQGLIFVVDCADRDRIDEARQELHRIINDREMRDAIILIFANKQDLPDAMKPHEIQ
Arf1 140 DKLGLHSLRHRNWYIQATCATSGDGLYEGLDWLSNQLRNQK
Arf2 140 DKLGLHSLRQRNWYIQATCATSGDGLYEGLDWLSNQLKNQK
Arf3 140 DKLGLHSLRHRNWYIQATCATSGDGLYEGLDWLANQLKNKK
Arf4 140 DKLGLQSLRNRTWYVQATCATQGTGLYEGLDWLSNELSKR
Arf5 140 DKLGLQHLRSRTWYVQATCATQGTGLYDGLDWLSHELSKR
Arf6 136 EKLGLTRIRDRNWYVQPSCATSGDGLYEGLTWLTSNYKS
B
Arf1 / EGFP-PTS1
Arf3 / EGFP-PTS1
Arf4-EGFP
Arf5 / EGFP-PTS1
Arf6 / EGFP-PTS1
- / EGFP-PTS1
67
43
30
20
α-EGFP
Arf4-EGFP
EGFP-PTS1
α-Arf6
Arf6
C
H6-GST-Arf1
H6-GST-Arf3
H6-GST-Arf4
H6-GST-Arf5
H6-GST-Arf6
H6-GST-DCOH
α-H6
α-Arf6
H6-GST-Arf
H6-GST-DCOH
H6-GST-Arf6
